# Serious hazards of transfusion: evaluating the dangers of a wrong patient autologous salvaged blood in cardiac surgery

**DOI:** 10.1186/s13019-022-01931-6

**Published:** 2022-08-16

**Authors:** Masashi Uramatsu, Hideyuki Maeda, Shiro Mishima, Megumi Takahashi, Jun Wada, Kagehiro Amano, Paul Barach, Tamotsu Miki

**Affiliations:** 1 Department of Quality and Patient Safety, Tokyo Medical University, 6-7-1 Nishi-shinjuku, Shinjuku-ku, Tokyo, 160-0023 Japan; 2 Section of Medical Safety Management, Tokyo Medical University Hospital, 6-7-1 Nishi-shinjuku, Shinjuku-ku, Tokyo, 160-0023 Japan; 3 Department of Forensic Medicine, Graduate School of Medicine, Tokyo Medical University, 1-1-1 Shinjuku, Shinjuku-ku, Tokyo, 160-8402 Japan; 4 Department of Laboratory Medicine, Tokyo Medical University Hospital, 6-7-1 Nishi-shinjuku, Shinjuku-ku, Tokyo, 160-0023 Japan; 5 Thomas Jefferson University School of Medicine, Philadelphia, PA USA; 6 University of Queensland School of Medicine, Brisbane, Australia; 7 Medical University of Graz, Graz, Austria

**Keywords:** Patient safety, Root cause analysis, Human factors, Autologous-salvaged blood, Transfusion, Wrong patient, Medical error

## Abstract

**Background:**

The past half century has seen the near eradication of transfusion-associated hazards. Intraoperative cell salvage while widely used still poses significant risks and hazards due to human error. We report on a case in which blood collected from a patient with lung cancer was mistakenly administered to a patient undergoing cardiac surgery who should have received his own collected blood. The initial investigation found that the cause of the patient harm was violations of procedures by hospital personnel. A detailed investigation revealed that not only violations were the cause, but also that the underlying causes included haphazard organizational policies, poor communication, workload and staffing deficiencies, human factors and cultural challenges.

**Case presentation:**

On August 14, 2019, a 72-year-old male was admitted to our hospital for angina pectoris and multivessel coronary artery disease. Cardiac surgery was performed using an autologous salvage blood collection system, and there were no major problems other than the prolonged operation time. During the night after the surgery, when the patient’s blood pressure dropped, a nurse retrieved a blood bag from the ICU refrigerator that had been collected during the surgery and administered it at the physician's direction, but at this time neither the physician nor the nurse performed the required checking procedures. The blood administered was another patient's blood taken from another surgery the day before; an ABO mismatch transfusion occurred and the patient was diagnosed with DIC. The patient was discharged 65 days later after numerous interventions to support the patient. An accident investigation committee was convened to analyze the root causes and develop countermeasures to prevent a recurrence.

**Conclusion:**

This adverse event occurred because the protocol for intraoperative blood salvage management was not clearly defined, and the procedure was different from the standard transfusion practices. We developed a new workflow based on a human factors grounded, systems-wide improvement strategy in which intraoperative blood collection would be administered before the patient leaves the operating room to completely prevent recurrence, instead of simply requiring front-line staff to do a double-check. Implementing strong systems processes can reduce the risk of errors, improve the reliability of the work processes and reduce the likelihood of patient harm occurring in the future.

## Background

Autotransfusion of patient blood has been widely used in various surgeries since the early 1970s [1]. One method of blood salvage is to collect the blood from the operative field and store it intraoperatively [2]. Intraoperative cell salvage is effective for the conservation of red blood cells [3]. Although technical errors in intraoperative cell salvage have been reported, there have been no reported cases of blood transfusion to a wrong patient, a so called "never event" [4]. In this case report, we describe a wrong patient blood infusion case that resulted in the wrong blood unit given to the patient collected as autologous-salvaged blood (ASB) during cardiac surgery. We investigated the causes of this never event using root cause analysis (RCA) approach to intuitively lay out the incident information and quickly show the cause-and-effect relationships that contributed to the patient harm, in order to prevent similar incidents.

## Case presentation

A 72-year-old Patient X with a history of congestive heart failure and chronic atrial fibrillation, requiring dialysis for chronic renal failure due to nephrosclerosis, was admitted to our hospital because of angina pectoris and multi vessel coronary artery disease. The patient’s home medications included warfarin potassium (1 mg), clopidogrel sulfate (50 mg), amiodarone hydrochloride (100 mg), and bisoprolol fumarate (1.25 mg). The patient’s blood type was O RhD positive. The blood was collected by the ASB system and the coronary artery bypass surgery (CABG) was successfully completed. The CABG surgery was prolonged due to a lenghty re-anastomosis of one of the three-vessel cardiac grafts. The operative time was 8 h 19 min, intraoperative blood loss was 790 ml, and no autologous or allogeneic blood transfusions were performed. The hospital usually stocks the collected blood in the intensive care unit (ICU) if the case does not require urgent use of the collected blood. The surgeon usually decides depending on the patient's condition whether to transfuse the blood back to the patient or discard it within 24 h after the operation.

The physician in charge on the night after the operation, decided to administer the salvaged blood of the patient due to a decrease in systolic blood pressure. The low blood pressure was attributed to dehydration as the patient was not bleeding, not in shock, and had no abnormalities in cardiac function, blood gases, or their electrocardiogram. The physician instructed nurse A to bring the patient’s blood from the ICU refrigerator. Because nurse A was engaged in the care of another patient, she requested that nurse B to “Bring it to me.” Nurse B went to the ICU refrigerator and picked up a blood bag. However, the bag retrieved by nurse B was not that of Patient X but was collected from another Patient Y, who had undergone thoracic surgery the day before to remove their lung cancer. The blood type of Patient Y was A RhD negative. When stored in the refrigerator, a note with the patient's name and identification (ID) number was supposed to be put on the basket, but this was not present at the time of the incident. The name was written on the pack, but nurse B did not check this because she was in a hurry. Nurse B believed that the blood in her hand was that of Patient X, and handed it directly to the nurse A, who then connected it to the patient's intravenous line and started the infusion without further ascertainment to asure the correct patient's blood identification. The two-person check at the bedside by nurses or physician and nurse was not performed. For a typical blood transfusion, a physician will use a Personal digital assistant (PDA) for verification, but the salvaged blood bag does not have a barcode and is not verified by a PDA.

The patient became hypotensive with blood pressures in the range of 50 to 60 mmHg systolic pressure range and vasopressors were initiated. Several minutes after the transfusion began, the patient's blood pressure dropped further, with a systolic pressure consistently in the low 50 s. The blood transfusion was stopped due to growing concern about the possibility of wrong blood transfusion. The volume of blood administered was estimated to be approximately 50 mL. The blood bag was removed from the venous line and the transfusion was stopped. Albumin and noradrenaline drop were administered, and over the next 15–20 min, the patient’s systolic blood pressure increased to the 130 mmHg range. There were no physical findings suggestive of an allergic reaction or hemolytic urine. The surgeon thought that the cause of the hypotension was a low circulating blood volume. One hour later, the patient’s serum hemoglobin level decreased to 9.2 g/dl from 11.4 g/dl preoperatively. The nurse was instructed to restart the transfusion. The nurse connected the blood that was hung on the bedside infusion table to the patient and restarted the transfusion. All residual blood was transfused into the patient. A decrease in the number of platelets was noted the next morning, blood pressures were lower and the patient was diagnosed with disseminated intravascular coagulation (DIC) syndrome.

The charge nurse discovered during a regular safety check of the ICU refrigerator that the ASB blood, which should have already been administered, was still stored. This revealed that the blood administered to Patient X was that of Patient Y. Patient X was successfully resuscitated and was discharged from the ICU on day 8. The patient was finally discharged home on day 65 due to persistent pleural effusions and positive CRP and had delayed rehabilitation due to severe back pain.

### Adverse incident aftermath: apology and disclosure

An hour after the accident occurred the concerned parties gathered to discuss the best course of action. Three and a half hours after the wrong transfusion was recognized, the surgeon in charge and staff of the patient safety department disclosed the facts of the events to the patient's family and apologized to the family [5]. Nine hours later, a second disclosure and apology were made to the patient's family. From day 12 to 60 day after the accident, several meetings and e-mail conferences were held involving the investigation committee, chaired by an external committee member tasked with analyzing the causes of the incident and proposing countermeasures to prevent a recurrence. The investigation report was submitted to the hospital President, who accepted the change recommendations. The hospital worked to be transparent during the investigation and issued statements about the changes being made to improve the reliability of their work processes and reduce the likelihood of a similar event occurring in the future. A periodic audit of blood transfusions indicated that all the recommended changes in ABS were implemented, and no other ASB related adverse events have occurred since.

## Discussion and conclusions

We describe a completely preventable wrong patient blood transfusion in which a patient was administered another patient’s blood, collected as autologous-salvaged blood (ASB) during cardiac surgery. We review the root cause investigation (RCA) and highlight the systems' issues that emerged and the corrective actions implemented in the hospital to prevent similar adverse incidents.

### Transfusion safety

The history of blood supply is one of early, sobering frequency of disease transmission but also remarkable improvement in systems safety in terms of correct blood transitions that is free of infectious agents. Blood product safety has been an improving area of focus over recent decades in many countries [6]. Several methods have been employed to reduce the risk of blood transfusions and improve blood product administration safety [7]. The entire blood harvesting and transfusion process has been redesigned from before blood donation collection through to the post-procedure follow-up with the blood product recipients. There have been improvements made in the collection, storage, management, distribution, utilization, and monitoring of transfusions.

### Transfusion system infrastructure

The transfusion service is one of the most highly regulated services within the clinical laboratory. The pathway of blood delivery is inherently complex because multiple patient care areas are involved. The goal of the blood delivery pathway is to deliver the right product to the correct patient [8].

The pathway can be summarized by three simple steps:

1. Identify the patient with two unique identifiers (ID).
2. Connect the patient identifiers to all prepared lab samples, tests, and blood products.
3. Deliver the right blood product to the right patient at the right time, confirming patient ID again.

These three simple steps comprise numerous processes, each with their own risks of failure, with the highest rates of failure associated with processes outside of the clinical laboratory.

### Risk of ABO-incompatible transfusions and hemolytic reactions

The risk of fatality due to an ABO-mismatched red blood cell transfusion is estimated at 1 to 4 per 10,000,000 of each red blood cell unit transfused [9]. But fatal reactions represent the ‘tip of the iceberg’ as most ABO-incompatible near miss transfusions involve small volumes due to early clinical signs/symptoms and rarely do patients not survive [10].

The risks of a lethal hemolytic transfusion reaction were estimated at 1 per 550,000 units transfused for the time period 1976–1985 in the US [11]. Not all hemolytic reactions are ABO-related and not all wrong transfusion events result in adverse clinical outcomes. Others have estimated that 1 in every 19,000 units of red blood cells is transfused to the wrong patient each year, 1 in 76,000 transfusions results in an acute hemolytic reaction, and, 1 in 1.8 million units of transfused red blood cell units results in death due to acute hemolytic reaction [12].

When estimating the risks, the best information available indicates that most transfusions to the wrong patient occur as a result of potentially avoidable system failures [13]. The most frequent error leading to transfusion of ABO-incompatible blood occurs during patient identification/verification at the bedside; as a result, although the blood is labeled appropriately, it can result in the wrong blood being given to the patient.

### Root cause analysis of the case (RCA)

**Fig. 1:**
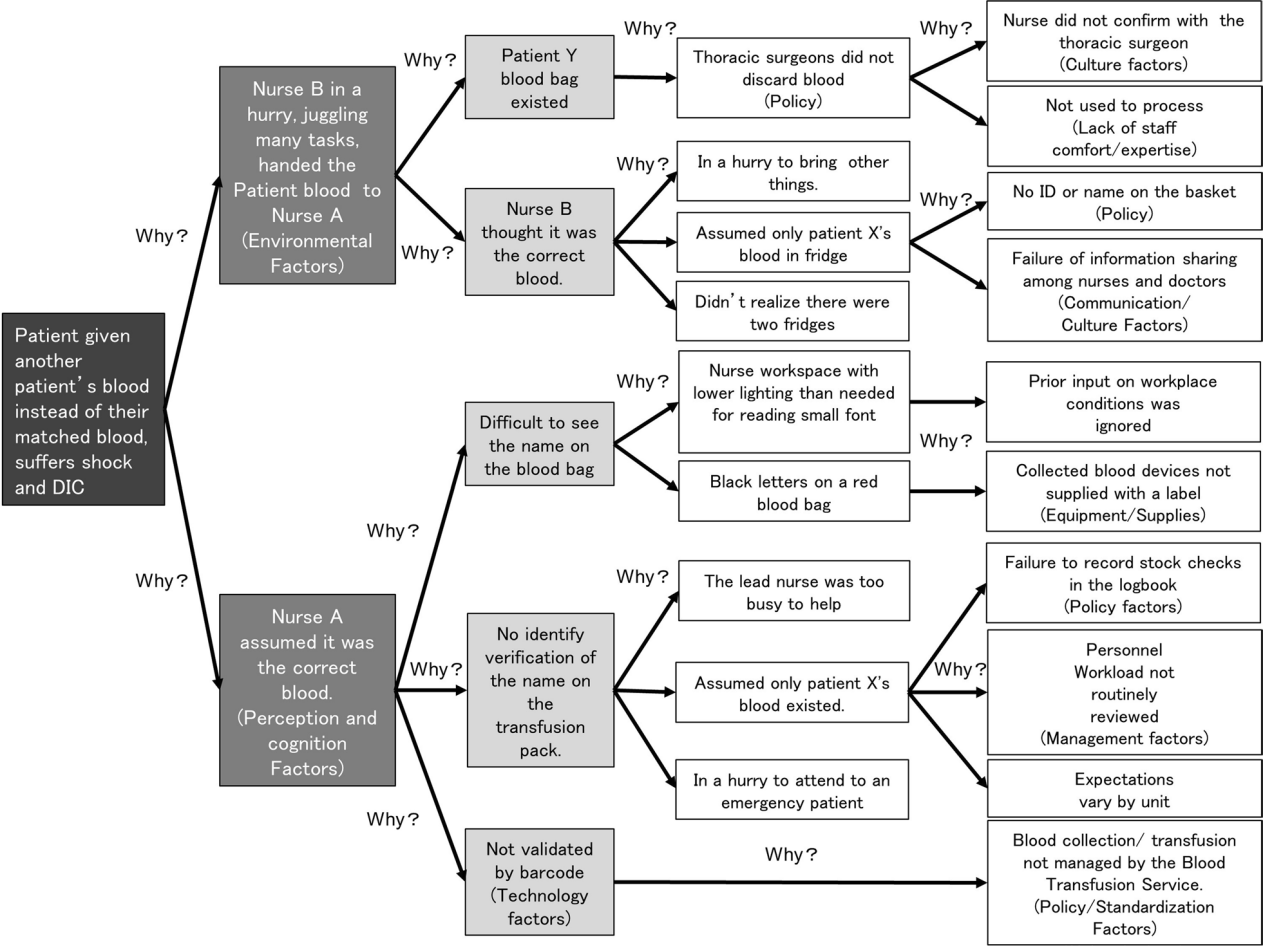
The linkage diagram. The linkage diagram provides a visual depiction of the contributory factors and underlying causes leading to the adverse event in this case. Two factors, human and environmental factors, are involved in Nurse A's behavior. The unique actions of Nurse A are shown on the bottom, and the environmental factors are shown on the top

A linkage diagram (Fig. 1) was created and a root cause analysis of events (Table 1) was developed to link the problem statement to the conditions and actions. An RCA, is a visual format for performing a root cause analysis, allowing us to intuitively lay out the information to quickly show the cause-and-effect relationships that contributed to this adverse incident. These charts help identify a number of factors that predispose the blood transfusion system to errors and revealed multiple contributing factors, including communication problems, human factors problems, inadequate policies and procedures, cultural problems, etc [14]. The downstream effects of the errors led to the wrong blood reaching the patient’s bedside and to a serious patient outcome. The linkage diagram does not contain all of the information, but it provides an overview of how the incident occurred and helps to organize the information in a way that can be quickly and visually understood.

**Table 1:** Root cause analysis of events and its application to understanding this case

Root cause	Application to the case
Communication problems	The blood salvaged during operation of Patient Y was not communicated within the Cardiovascular Surgery department, Thoracic surgery department and among the nurses. Most of them were unaware that Patient Y's blood was being stored postoperatively. Errors occurred in perception of the risks and the cognition regarding how to avoid this error. Patient Y's blood was not discarded on POD 1 as hospital policies dictate, nor was its presence shared among the nurses or even within the Cardiovascular Surgery department.
Inadequate information flow	Instructions for blood salvaging were not clearly communicated either verbally or in writing. Nurses did not communicate with other nurses or physicians about their concerns regarding Patient Y's condition and questions regarding blood salvage protocols.
Human factors problems	Since collected blood devices were not equipped with a dedicated label, the patient's name was written directly on the red transfusion bag with a black magic marker. In addition to this, Nurse B could not recognize that the blood was from the wrong patient because the intensive care unit in the evening was dimly lit and the visibility was poor. Nurse A, nurse in charge of Patient X Nurse B, the nurse in charge of Patient Y, to retrieve the blood in the cold storage, and was in a hurry because the patient's condition was unstable.
Patient-related issues	The patient was transferred to the ICU after a lengthy surgery, and their blood pressure was unstable.
Organizational transfer of knowledge	Nurse B did not know that the Intensive Care Unit had two cold refrigerators for storing blood.
Staffing patterns/work flow	The reason for this is that the intensive care unit was always busy, and the duties of the lead nurse were shared among several staff members. The division of duties was the reason why labels were not applied, blood was not checked per hospital policies, and entries were not made in the logbook, nor was their absence noticed. However, if the patient waunt stable, and providers had more time to check the blood, they could have correctly identifred the error and followed the hospital policy. Multiple healthcare provider teams were involved in the care of the patents, which also contributed to the communication challenges,
Technical failures	Both the physicians and nurses on site assumed that the only blood collected was from Patient X. They did not know that Patient Y's blood was stored. Therefore, they connected the Patient Y blood to Patient X's IV line, and administered it without doing the necessary checks.
Inadequate policies and procedures	The Surgery department did not issue an order to discard the blood on the following day. This was due to the lack of a written procedure and the unfamiliarity of the Thoracic surgeons with ABS hospital policy. Intraoperative salvaged blood should have been placed in a dedciated basket with a note attached with the patient's name and ID, and placed in cold storage. The blood in the cold storage was supposed to be checked twice a day by the lead nurse and recorded in the management log. However, there was no record of these activities. It is against hospital policy to salvage blood products in cold storage where the temperature is controlled by the Blood Transfusion Service. The Blood Transfusion Service was unaware that intraoperative blood collections were kept away from patients and stored in cold storage. Therefore, the operating room and intensive care unit were unable to assess and verify the blood collection procedures of the operating room and intensive care unit. The Blood Transfusion department could not question the blood salvage procedures in the operating room and intensive care unit because they were unaware that intraoperative salvaged blood were kept away from the patient and stored in cold storage. The central operating department and intensive care units were in a position to correct such misuse, but they did not have written procedures for handling intraoperative blood collection and did not exercise proper governance and oversight. The basket containing Patient Y's blood did not have a note attached with the name and ID, and the pack did not have a dedicated label.

Patient X: Patient underwent cardiac surgery. Blood type was O, Rhesus (Rh) D-positive. Patient Y: Patient underwent lung surgery. Blood type was A, Rhesus (Rh) D-negative. POD: Postoperative day. ICU: Intensive Care Unit. ID: Identification; POD-Post operating day

## Missed opportunities

Unfortunately, but importantly, the linkage diagram shows the patient’s adverse event was not the result of a single error. Most errors are caused by a combination of cascading failures, and it is rare that a single error leads to an incident. Initially, the cause of this accident appeared to be a simple case of rule violation, as the blood was administered by a nurse without checking the name against the blood bag label [15]. However, upon deeper reflection, it became clear that identifying this as the cause and taking measures to prevent recurrence, such as double-checking the patient details, may not have prevented a recurrence [16–18] in the future. Therefore, the investigation committee searched for system errors at a deeper level. There were multiple process failures and at several missed opportunities where this error could have been either prevented or caught earlier.

Nurse B's action in "administering the wrong infusion" was considered a possible cause of human error, which was investigated using the RCA (see below). Human error is defined as the failure of planned actions to achieve their desired ends without the intervention of some unforeseeable events, and it is not the cause of the accident; it is the factor that causes the human to make the error that is the cause of the accident [19]. Human behavior is the result of a combination of human and environmental factors, known as the *Lewin’s equation* [20] or *the SHELL model* [21]. Figure 1 shows an extract from the RCA. Two factors, human and environmental factors, are involved in Nurse A's behavior. The unique actions of Nurse A are shown on the bottom, and the environmental factors are shown on the top of the figure.

First, we consider the human factors involved [22]. We explored the reasons why Nurse A believed that the blood bag in her hand belonged to Patient X and not to Patient Y. The probable background factors included that the: (1) autologous salvaged blood units from multiple patients were stored in a single refrigerator in the ICU and this information was not shared among the staff; (2) the patient’s ID and name were handwritten on the bag after collection, not labeled as required; and (3) barcode matching system was not applied to the salvaged blood and the ABS administration was not managed by the Blood Transfusion Service.

Next, the environmental factors were discussed. The cause of Patient Y's blood being passed into the hands of Nurse A as Patient X's blood was discussed. The probable background factors for how Patient Y's blood got into the hands of Nurse A were as follows: (1) the blood collected from multiple patients was stored in multiple refrigerators in the ICU; (2) the patient’s name and other information were not written on the basket as required when storing the blood bag in the refrigerator; (3) normally, a paper label with the patient's name and ID number must be affixed to the bag after blood collection before usage, but in this case, this label was not used, and the patient's ID number and name were hand written directly on the transfusion bag with a black magic marker; and (4) the thoracic surgeon who salvaged the blood from Patient Y was not familiar with blood salvage protocols and did not make a decision within 24 h to use or discard the blood.

It is clear from considering factors 1–3 above, that there were no clear SOPs defined in the ICU. Nurse A administered the incorrect blood unit, believing that the blood unit in her hand belonged to Patient X, when in fact it belonged to Patient Y. The investigation committee determined that the "intraoperative salvaged blood, without starting to administer the blood before leaving theatre, and bringing the bag to the ICU," was the lead root cause of this event.

### Corrective and preventative action plan

The investigation committee believed that measures addressing the violations of the confirmation process by the staff, background factors such as lack of information sharing, and compliance failures in storage and destruction of bags, while critical, would not have completely prevented this from recurring. Therefore, measures to prevent a recurrence were focused on the upstream causes, which includes how procedures for intraoperative salvaged blood are managed. Transfused salvaged blood is generally recommended to be administered in the operating room but it is not prohibited to be given outside the operating room. However, to prevent blood unit mix-ups, it is necessary to start the ABS administration in the operating room. The draft guidelines for the Implementation of Transfusion of Autologous Salvaged Blood (2020) by the Japanese Society for Autologous Blood Transfusion indicates that “in principle,” administration should be started in the operating room [23]. At our hospital, we decided to go one step further and require starting all ASB administration in the operating room without exception. Additionally, new regulations have been established regarding how to record blood salvage, label ABS bags, and instructions for administration and disposal, which previously were not clearly stated. These regulations will be managed by the Blood Transfusion Service of the hospital. Cell salvage equipment and staff trained are needed to operate immediately and be available 24 h a day when undertaking surgery where blood loss is a potential complication. The hospital should nominate a clinical lead and a coordinator for cell salvage, who oversee a competence-based training program for all involved staff, along with ongoing data collection and regular data audits. This training should be overseen by the Blood Transfusion Division.

In conclusion, we investigated a serious adverse patient incident in which intraoperatively salvaged blood was transfused to the wrong patient with a different blood type. Under the Human Factors  theory, failures are not satisfactorily explained by demonstrating human deviation from expected behavior. Instead, the circumstances and underlying pressures are meticulously explored, and systemic deficiencies are identified. It is always easier to see the warning signs in hindsight but in reviewing incidents of missed warning signs we are reminded to look for patterns of smaller incidents and to take near-misses seriously. Interventions to improve blood safety should lean towards design improvements, engineering controls, or process simplification and standardization. We developed and implemented a systems' improvement strategy that requires a stringent verification process in collecting and labeling blood specimens. The intraoperative blood collection is administered before the patient leaves the operating room in order to completely prevent a wrong blood administration recurrence, instead of simply requiring front-line staff to check or double-check the blood compatibility. The hospital has been working to improve the reliability of their work processes, enhancing policy and training improvements, and making all efforts to reduce the likelihood of a similar error occurring in the future. While strict rules are a reminder of what not to do, people’s ability to perceive the situation and adapt is the primary source of safety.

## Data Availability

The datasets used and/or analysed during the current study are available from the corresponding author on reasonable request after approval by our Ethics Committee.

## Abbreviations

ASB: Autologous Salvaged Blood
RCA: Root Cause Analysis
Rh: Rhesus
ICU: Intensive Care Unit
ID: Identification
PDA: Personal Digital Assistant
